# Photoprotection of *Buddleja cordata* extract against UVB-induced skin damage in SKH-1 hairless mice

**DOI:** 10.1186/1472-6882-14-281

**Published:** 2014-08-03

**Authors:** José Guillermo Avila Acevedo, Adriana Montserrat Espinosa González, Diana Matamoros De Maria y Campos, José del Carmen Benitez Flores, Tzasna Hernández Delgado, Saul Flores Maya, Jorge Campos Contreras, José Luis Muñoz López, Ana María García Bores

**Affiliations:** Laboratorio de Fitoquímica, Unidad de Biología, Tecnología y Prototipos (UBIPRO), Facultad de Estudios Superiores-Iztacala, Universidad Nacional Autónoma de México, Av. de los Barrios No. 1, Los Reyes Iztacala, Tlalnepantla, Edo. de México C.P. 54090 México; Laboratorio 1, UMF, Facultad de Estudios Superiores-Iztacala, Universidad Nacional Autónoma de México, Tlalnepantla, 54090 Edo. de México México; Laboratorio de Farmacognosia, UBIPRO, Facultad de Estudios Superiores-Iztacala, Universidad Nacional Autónoma de México, Tlalnepantla, 54090 Edo. de México México; Laboratorio de Recursos Naturales, UBIPRO, Facultad de Estudios Superiores-Iztacala, Universidad Nacional Autónoma de México, Tlalnepantla, 54090 Edo. de México México; Laboratorio de Bioquímica Molecular, UBIPRO, Facultad de Estudios Superiores-Iztacala, Universidad Nacional Autónoma de México, Tlalnepantla, 54090 Edo. de México México

**Keywords:** *Buddleja cordata*, Phenolics, Antioxidant, UV radiation, Sunburn, Erythema, Photoprotection

## Abstract

**Background:**

In recent years, there has been considerable interest in using botanical agents to prevent skin damage resulting from solar UV-irradiation. *Buddleja cordata* is a plant that is known as “tepozan”. Some people in Mexico use the leaves of this plant to treat tumours, abscesses, sores and burns. The purpose of this study is to investigate the photoprotective properties of *Buddleja cordata* methanolic extract (BCME) against UVB-induced skin damage in SKH-1 hairless mice at the macroscopic and histological levels.

**Methods:**

BCME was characterised to determine its spectroscopic, chromatographic and antioxidant (DPPH, superoxide and hydroxyl radicals) properties. To conduct the photoprotection studies, BCME was applied topically to the skin of SKH-1 mice before acute exposure to UVB for 10 minutes. The murine skin samples were used for macroscopic and histological studies to assess tissue damage. Penetration of active components of BCME into stratum corneum on the dorsal area of mice was investigated *in vivo* by the tape stripping method. Moreover, genotoxicity of BCME was evaluated in a *Vicia faba* cell root micronucleus model.

**Results:**

BCME displayed absorbance over the entire UVB spectrum, and its principal components included verbascoside and linarin. BCME exhibited antioxidant activity and significantly scavenged hydroxyl radicals. BCME reduced erythema, sunburn cell production, vessel congestion and epidermal thickening of UVB irradiated mouse skin. BCME penetrate the skin of mice. BCME did not exhibit genotoxic activity in the micronucleus test.

**Conclusion:**

The topical administration of BCME protected against acute UVB-induced damage in mouse SKH-1 skin, and our results suggest that BCME may potentially prevent photodamage.

## Background

The sun generates various types of radiation via thermonuclear reactions, including ultraviolet radiation (UVR). The amount of UVR that crosses the atmosphere and reaches the earth’s surface is approximately 6% of the total radiation produced by the sun. The UV spectrum can be divided into UVC (200–280 nm), UVB (280–320 nm) and UVA (320–400 nm) wavelengths. In the earth’s atmosphere, ozone (O_3_), oxygen (O_2_) and water vapour (H_2_O) selectively filter both UVC and UVB radiation. Due to this phenomenon, UVA comprises approximately 95% of the UV radiation that reaches the earth’s surface. Currently, almost no UVC radiation penetrates the atmosphere, and approximately 90% of the UVB radiation is absorbed by the ozone layer, depending on the geographic location and time of the day. Because the ozone layer has been depleting by the effects of chlorofluorocarbon (CFC) pollution, more UVB and even some UVC radiation can now reach the earth’s surface [1]. UVB has previously been identified as the primary etiological agent for the development of non-melanoma and melanoma skin cancers. UVB can act as both initiator and promoter of tumours by damaging cellular macromolecules such as DNA. When absorbed by the skin, UVB is able to induce photochemical damage by forming cyclobutane pyrimidine dimers and pyrimidine (6–4) pyrimidone photoproducts in the DNA of epidermal cells. These photoproducts are the predominant pre-mutagenic events responsible for the initiation of non-melanoma cancers of the skin [2]. DNA damage leads to the altered expression of various gene products involved in protective or reparative processes, such as apoptosis, genomic repair and cell growth arrest. Deregulated sunburn apoptosis, together with the loss of normal function of the product of the p53 gene and immunosuppression, also appear to play an important role in photocarcinogenesis [3]. UVB may initiate the activation of many reactive oxygen species (ROS)-sensitive signalling pathways, thereby causing an increase in the cellular levels of ROS [4]. This increase in oxidative stress results in an imbalance between ROS and the skin’s endogenous antioxidant defence system and results in damage to important molecular structures in the cells of the skin, such as DNA, proteins and lipids [5]. Acute exposure to UVB induces sunburn, hyperpigmentation, hyperplasia and inflammatory processes in the skin, including erythema, edema, pain and heat [6]. In recent years, there has been considerable interest in using botanical agents for the prevention of skin damage upon exposure to solar UV-irradiation. The most promising chemical substances are members of the polyphenol family. Recent studies indicate that these substances are photoprotective due to their antioxidant and sunscreen properties. Photochemoprevention, which is defined as the use of agents capable of ameliorating the adverse effects of UVB on the skin by natural compounds, represents a new concept in the attempt to control the process of carcinogenesis. Many photochemoprotective agents have recently been identified from botanical origins and hold promissory value to combat UVR exposure [7].

*Buddleja cordata* H.B.K. ssp. cordata (Buddlejaceae) is a shrub or tree that is widespread throughout Mexico [8]. This species grows in quercus, conifers and cloud forests and as secondary vegetation. Because the species is tolerant to drought conditions, it is also found in xerophytic shrublands and eroded soil. This plant is distributed from northern Mexico to Guatemala at altitudes between 1500 and 3000 meters above sea level [9].

In Mexico, *B. cordata* is also known as “tepozan”, “matowi” or “wasala”, and this plant has been employed for many uses since pre-conquest times. Its use was documented in the Badian codex written by Martin de la Cruz in the 16th century [10]. According to this source, the natives of Mesoamerica used this plant for the treatment of “mentagra” or “chinwelk”. In recent times, people have utilised its leaves as a poultice to treat tumours, abscesses, sores and burns. Decoctions of the leaves, roots and bark are used as a diuretic that is administered orally and applied topically to heal wounds and rheumatic pains [11]. In northern Mexico, the Raramuris (Tarahumara) Indians utilised the wood and bark for treating skin ailments and inflammations as well as for ritualistic medicinal purposes or cultural diseases, such as fright and soul loss [12]. People in Mexico currently use *B. cordata* empirically for the treatment of diseases with cancer symptomatology. Today, such treatments require the use of scientific studies [13].

Previous chemical studies of *B. cordata* have led to the isolation and identification of bioactive flavonoids [14, 15], antifungal sesquiterpenoids [16], anti-*Mycobacterium* phenylethanoids [17] and antibacterial phenylpropanoids [18].

Multiple studies have demonstrated the high capacity of several plant polyphenolic compounds (such as flavonoids and hydroxycinnamic acids) to protect living organisms from alterations induced by UV radiation, including skin damage [7]. In particular, there is evidence supporting the notion that phenylpropanoids and flavonoids are endowed with strong photoprotective properties and can efficiently protect skin cells against the deleterious effects of UV light, either as pure molecules or as polyphenolic plant extracts [19].

In a previous research study, we evaluated the photoprotective activity of *Buddleja scordioides* extract and its compounds using bacteria and guinea pigs as experimental systems [20]. The current study was designed to investigate the photoprotective effects of *Buddleja cordata* methanolic extract (BCME) against UVB-induced skin damage in SKH-1 hairless mice at the macroscopic and histological levels. Moreover, the antioxidant and genotoxic properties of BCME were also evaluated.

## Methods

### Plant material

*B. cordata* leaves were collected in the forested areas of Pedregal de San Angel, near the campus of the Universidad Nacional Autónoma de México (UNAM). The campus is located on the south side of Mexico City. The collection occurred in March 2010 and was authenticated by the corresponding author. A voucher specimen (No. 42663) was deposited at the Izta Herbarium of the Universidad Nacional Autónoma de México. The plant material was air dried indoors at room temperature before extraction.

### BCME preparation

Leaves of *B. cordata* (253 g) were dried, ground and extracted with hexane and methanol in succession. The methanolic portion was evaporated under reduced pressure at 55°C to obtain a syrup. The resulting dry residue (29.55 g) was stored at 4°C and is hereafter referred to as *Buddleja cordata* methanolic extract (BCME).

### UV absorption analysis of BCME

The UV absorption spectrum of BCME was obtained using a UV/VIS spectrophotometer (Perkin-Elmer, Lambda 2S UV/VIS). BCME and the commercial organic filter octyl methoxycinnamate (Parsol, ISP VAN DIK) were dissolved in ethanol at a concentration of 60 μg/mL and 10 μg/mL respectively. The samples were placed in a standard quartz cuvette with a 1 cm path length and then quickly scanned with UV light in the range of 200–400 nm.

### HPLC analysis of BCME

The HPLC system consisted of a Hewlett Packard Series 1100 HPLC instrument with a UV detector set at 320 nm. The column was obtained from Supelco Technologies C18 (150 mm × 4.6 mm, 5 μm). The eluent was a mixture of 4% tetrahydrofuran in acetonitrile and water (35:65, v/v) and contained 0.04% phosphoric acid. The flow rate was 1 mL/min, the column temperature and pressure were 23°C and 149 bar, respectively, and the injection volume was 20 μL. Verbascoside, linarin and syringin served as standards.

### Determination of the total polyphenol content of BCME

The total polyphenol content of the methanolic extract was determined using Folin Ciocalteu’s phenol reagent according to the method described previously [21]. The total polyphenols were expressed as mg of gallic acid equivalents per gram of extract.

### *In vitro*antioxidant assays

Scavenging of DPPH free radicals was measured using a modification of Lohézic-Le Dévéhat method [22] in which BCME (5–300 μg/mL), gallic acid (0.5-10 μg/mL) and DPPH (250 mM) were dissolved in methanol. At least six different dilutions of extract were tested and allowed to incubate for 20 min in the dark before absorbance was measured at 517 nm in an ELISA lector spectrophotometer (Thermo Scientific, USA). The experiment was conducted in triplicate. The scavenging activity of each concentration was calculated as a percentage of reduction in DPPH concentration as follows:


Where Abs DPPH is the absorbance of the DPPH in methanol, and Abs Sample represents the absorbance of each sample in the presence of each concentration of BCME and gallic acid. Antioxidant activity was expressed as an IC_50_ value (inhibitory concentration in μg/mL of sample or positive control necessary to reduce the absorbance of DPPH by 50% compared to the negative control). The positive control was gallic acid. A lower IC_50_ value represents a higher antioxidant activity.

Scavenging of superoxide radical (O_2_^•^-) was measured [22] using 96-well microplates and a non-enzymatic technique. The reaction mixture in the sample wells consisted of NADH (78 μM), nitro-blue tetrazolium (NBT) (50 μM), phenazine methosulfate (PMS) (10 μM) and BCME (500, 250, 125, 62.5, 31.25 μg/mL). The reagents were dissolved in 16 mM tris-hydrochloride buffer (pH = 8), except for BCME, which was dissolved in DMSO. After 5 min of incubation at room temperature, the spectrophotometric measurement (Thermo Scientific. USA) was performed at 560 nm against a blank lacking PMS and sample. Gallic acid was used as a positive control. The percentage inhibition at a steady state was used to calculate the IC_50_ values for each dilution. This measurement determined the amount of antioxidant required (measured as the concentration of the stock solution added to the reaction mixture) to scavenge 50% of O_2_^•^-, with the lowest values representing the best efficiency for scavenging O_2_^•^-. All tests were performed in triplicate, and the results were averaged.

Hydroxyl radical scavenging was measured according to the Halliwell method [23]. Hydroxyl radical (^•^OH) was generated by a Fenton system (ascorbic acid/FeCl_3_EDTA/H_2_O_2_). Deoxyribose (DR) is degraded to malonaldehyde when exposed to hydroxyl radicals, which generates a pink compound in the presence of thiobarbituric acid at low pH under heat. The reaction mixtures contained the following reagents at the indicated final concentrations (in a final volume of 1 mL): potassium phosphate buffer, pH 7.4 (10 mM), DR (2.8 mM), H_2_O_2_ (1.42 mM), BCME at different concentrations, FeCl_3_–EDTA (20 and 100 μM) and gallic acid was used as a positive control. The iron salt was premixed with the chelator and dissolved in water before addition to the reaction mixture. All other components were dissolved in potassium phosphate buffer, pH 7.4 (10 mM). After incubation at 37°C for 1 h, 1 mL of 2.8% (w/v) trichloroacetic acid and 1 mL of 1% (w/v) TBA were added, and the mixture was heated in a water bath at 100°C for 15 min. The absorbance of the resulting solution was measured at 532 nm.

### Experimental animals and protocol

The SKH-1 hairless mouse strain, a widely used model for human photocarcinogenesis, was used in our study. Female SKH-1 mice at 5–6 weeks of age (weighing 26 ± 5 g) were purchased from Charles River Laboratories (Wilmington, MA) and maintained in a climate-controlled environment with a 12 h light/dark cycle. Five mice were housed per cage and acclimatised for two weeks before starting the experiment. Throughout the experimental period, mice had free access to food and water that were provided through the food chamber on top of the cages. The Bioethical Committee/FES Iztacala, UNAM approved all animal protocols.

The SKH-1 mice were randomly divided into five groups of five mice each: untreated (U), negative control (C-), and positive control with UV (C+UV)*,* BCME and BCME irradiated with UV (BMC+UV).

The mice in the negative and positive control groups were treated topically on the dorsal skin with 200 μL of ethanol (exposed area: 6 cm^2^). BCME was dissolved in ethanol at a concentration of 2 mg/mL [20]. The BCME groups were treated topically on the dorsal skin with 200 μL of the respective test solutions.

Mice that were untreated and pre-treated with ethanol (without UVB irradiation) were used as the normal and negative controls for analysis of normal skin, respectively. A treatment with ethanol and UVB irradiation served as the UVB-induced skin damage positive control. Treatments with BCME served as the experimental groups (without UVB and with UVB irradiation). Fifteen minutes after application of the substances, the C+UV and BCME+UV groups were irradiated with UV-B lamps (302 nm, UVP. UVM-26, 6 W) positioned 15 cm above their backs. Irradiation at this distance produced a dose of 6 mJ/cm^2^, which was measured with a Spectroline model DM-300HA research radiometer. The irradiation-exposure time in acute experiments was ten minutes. After 24 h of irradiation, erythema was measured. Animals were subsequently sacrificed by carbon dioxide asphyxiation, and the dorsal skin was dissected.

### Erythema measurements

The erythema in mice was calculated by measuring the skin redness of the dorsal area of mice using a colour analyser (Lutron, Mod. RGB-1002). Among the various methods, colourimetry has the advantage of being the simplest and most reproducible method. Redness values were measured 24 hours after irradiation. The results are expressed as redness values for each treatment [24].

### Histological observation of the skin

Each dorsal skin sample was fixed with 2% paraformaldehyde in phosphate buffer solution (pH 7.2) for 24 h in the tissue embedding cassette, dehydrated with a sequence of ethanol solutions (70%, 80%, 95% and 100%, v/v) and embedded in paraffin. All serial sections were cut to a thickness of 5 μm, de-paraffinised, and stained with haematoxylin-eosin (HE). The histopathological changes of each section were observed using multiple microscopic fields and photographed with a photomicroscope (Nikon). Measurements included the epidermal thicknesses and the number of sunburned cells (400X). The histological diagnosis was performed comparing the untreated and C- mouse skin samples with UVB irradiated groups in accordance with the number of sunburns reported [25].

### *In vivo*BCME penetration study: tape stripping

Female SKH-1 mice at 5–6 weeks of age (n=5, weigh on 26 ± 5 g) were placed in a laminar flux chamber at 21°C and 62% relative humidity for 30 min. Application zones measuring 2 × 2 cm^2^ was marked for each mouse in dorsal area. The BCME was applied to the surface of the skin at 2 mg/cm^2^. 15 min after an application, the excessive substance on the applied area was wiped off with a cotton swab, followed by washing with ethanol 96% and drying. Stratum corneum of the treated areas was removed by four successive tape strip-pings using Scotch tape strips (19 mm × 32.9 m, 3 M, MN, USA). Each strip was taken in a controlled way, i.e. a 10 g rubber weight was rolled over it 10 times. The first strip was discarded and the three following strips were collected and deposited in a 20 mL beaker. 5 mL of ethanol were added at each sample and then stirred with a magnetic bar for 30 min. The absorbance was measured at 320 nm using a double-beam spectrophotometer (Perkin-Elmer, Lambda 2S UV/VIS) because the BCME main components absorb that wavelength. BCME penetration was obtained in accordance with concentration measurements calculated by regression analysis [26, 27].

### Genotoxicity assay: micronucleus test

Genotoxicity was determinate according to the Ma method [28]. Seeds of *Vicia faba* var. minor that had been previously stored at 4°C were used for this study. Dry seeds of *Vicia faba* were soaked for 24 h in distilled water, the seed coats were removed, and the seedlings were allowed to germinate between two layers of moist cotton. After seven days, the primary roots (approximately 1–2 cm in length) were selected randomly, and 4 seeds were used per treatment. Growing roots were treated for 2, 4, or 8 h with BCME at various concentrations (0.1, 0.2 and 0.4% w/v), followed by a 44 h recovery period. Tap water and 1% glycerine were used as negative controls. The exposure time was 48 h for the negative controls. Each batch was incubated at 22°C. At the termination of the exposure time for each treatment, the roots were cut to approximately 0.5 cm and placed into a Farmer solution (1:3 acetic acid and absolute methanol) at 4°C for 24 hours. The roots were subsequently hydrolysed in 1 N HCl at 55°C to break the bonds of the plant cell wall. The samples were rinsed three times with distilled water to remove the HCl and placed in 70% alcohol at 4°C. The roots were macerated in 45% acetic acid and were then stained for 5 min with 1% orcein acetate for observation via an optical microscope (Nikon). Three thousand cells per experimental group were scored to determine the mean frequency of micronuclei and the mitotic index. Each experiment was repeated three times. To calculate genotoxicity, the following equations were used: Mitotic Index (MI) TOTAL = number of cells from each phase × 100/1000 and Micronuclei (MCN) = Number of interphase cells with MCN × 100/1000.

### Statistical analysis

A statistical analysis was performed on the collected data. The mean values of the negative and positive controls and the BCME groups were obtained from descriptive analyses, and a One-Way ANOVA test was conducted to obtain *F* values and MS errors. Dunnett’s test was used to determine the level of significance in comparison to the negative and positive control values in each experimental series.

## Results and discussion

### BCME characterisation

**Figure 1:**
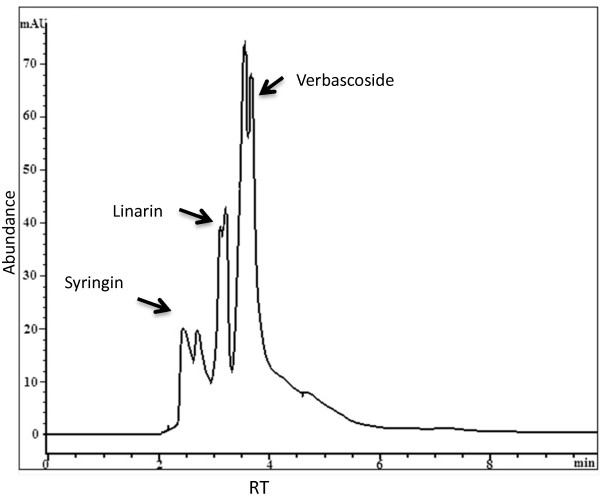
**High-performance liquid chromatography–ultraviolet (HPLC–UV) chromatogram of** ***B. cordata*** **methanolic extract (BCME).**

**Figure 2:**
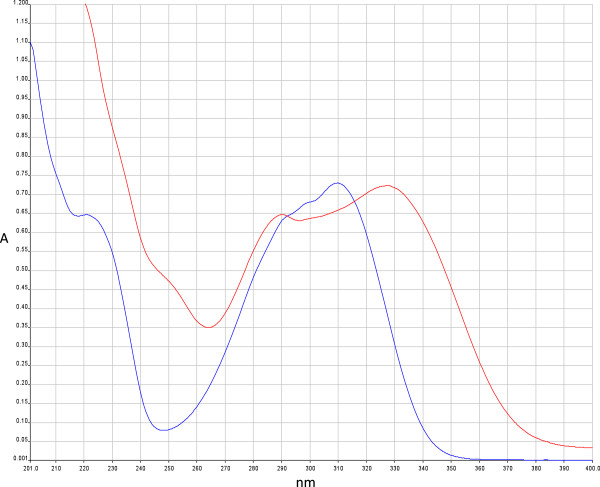
**UV absorption spectrum of** ***B. cordata*** **methanolic extract (BCME 60 μg/mL red line) and octyl methoxycinnamate (10 μg/mL blue line).**

The presence of phenolic compounds in BCME was quantified using the Folin-Ciocalteu test and yielded 177.13±1.97 mgEq gallic acid/g, which represented 17.71% phenolic compounds present in the extract (Table 1). The content of phenolic compounds in BCME is of the same order of magnitude as that reported for other species of the genus. Mahlke [29] obtained an ethanol extract of *Buddleja thyrsoides* containing 214.07 mg/g, while in Pan [30] an extract of *B. officialis* containing 113.56 mg/g. *Buddleja* genus plants synthesise phenolic compounds such as flavonoids and phenylpropanoids and are particularly abundant in linarin and verbascoside [14, 20]. Verbascoside and linarin have a strong absorption in the UVB region (291 and 334 λmax for verbascoside and linarin, respectively). Because verbascoside also exhibits antioxidant properties, we decided to quantify the content of these compounds in BCME. The results of the HPLC quantitative analysis of BCME are reported in Figure 1. The concentrations of both verbascoside and linarin were significantly high in BCME (98.75 mg/g and 36.45 mg/g, respectively). Syringin was also detected. An efficient photoprotective substance must have a strong absorption in the ultraviolet region to reduce the deleterious effects caused by exposure to sunlight. As observed in Figure 2, BCME exhibited three peaks in the UV region at 224, 290 and 324 nm. These data indicated that BCME absorbed all of the UV-B irradiation and thus displayed a sunscreen effect because the UVB irradiation is the primary cause of erythema. The results also indicated that BCME at 60 μg/mL exhibits similar UV absorption (Abs ≅ 0.7) than octyl methoxycinnamate at 10 μg/mL. BCME absorbance is significant since it is a mixture whose chromophores (linarin and verbascoside) are diluted in the extract.

**Table 1:** **Total phenolic content, antioxidant properties and penetration of** ***B. cordata*** **methanolic extract (BCME)**

Substance	Total phenols (mgEq gallic acid)	IC _50_ (μg/mL)			Penetration stripping test	
		DPPH	O _2_•-	HO•	%	μg/mL
BCME	177.13±1.97	64.19±2.09	133.60±35.20	1.85±0.10	0.82±0.2	33.40±9.0
Gallic acid	–	2.60±0.12	30.00±6.85	335.38±3.06	–	–

– Not determined.

### BCME *in vitro*antioxidant activity

Generally, exposure of skin to UV-B radiation results in increased of ROS generation, which contributes to several pathological conditions, including erythema, photocarcinogenesis and photoaging. The antioxidant capacity (IC_50_) of BCME extract was evaluated by DPPH, O_2_^•^- and ^•^OH radical scavenging assays. The IC_50_ values obtained for BCME scavenging effects for the three free radicals are shown in the Table 1. The ability of BCME to scavenge DPPH (IC_50_ 64.19 μg/mL) and superoxide (IC_50_ 133.60 μg/mL) radicals was lower than that of gallic acid (IC_50_ 2.60 and 30.00 μg/mL, respectively). The main antioxidant compound detected in BCME was verbascoside (≅10% of BCME weight). Frum [31] obtained an IC_50_ of 7.188 ± 0.12 for verbascoside activity against the DPPH radical. The IC_50_ for BCME is consistent with the concentration of verbascoside in BCME.

BCME exhibited excellent ^•^OH radical scavenging ability (IC_50_ 1.85 μg/mL) when compared with gallic acid (IC_50_ 335.8 μg/mL). Among the ROS, ^•^OH is the most reactive species in biological systems and is capable of damaging almost every molecule found in living cells [32], including lipids, polypeptides, proteins and DNA. From the resulting data (Table 1), it is notable that a lesser IC_50_ value was obtained for BCME when compared to the IC_50_ value of gallic acid (p<0.01). Possible additive or synergistic effects between verbascoside and any component of BCME could explain the observed anti-oxidant IC_50_ value of the crude plant extract.

### BCME anti-erythema activity

**Figure 3:**
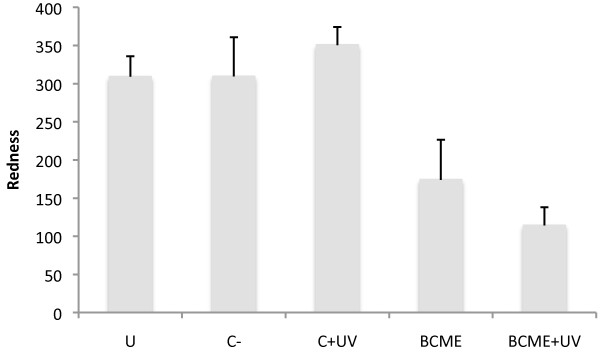
**Colorimetric values of erythema (redness) in SKH-1 mice.** U: group untreated; C-: Negative control (Vehicle 200 μL of ethanol); C+UV: Vehicle and UV; BCME: *B. cordata* methanolic extract; BCME+UV: *B. cordata* methanolic extract and UV.

One of the principal events in acute skin UVB irradiation exposure is the production of erythema (reddish skin). Erythema is visible 24 hours after exposure to UVB. As shown in Figure 3, irradiated unprotected SKH-1 mice exhibited increased skin redness within 24 h of UV exposure. The batches of mice treated with BCME showed less redness than normal, as did the negative and vehicle control groups.

For erythema, the determining factor of skin colour is the extent to which vascular filling occurs, where an increase or decrease in filling results in a greater degree of redness or paleness of the skin, respectively [24]. The presence of sunscreen substances in BCME would cause these differences, as would the presence of compounds exhibiting vasodilator properties, which could also account for the paleness effect.

### Histological analysis

**Figure 4:**
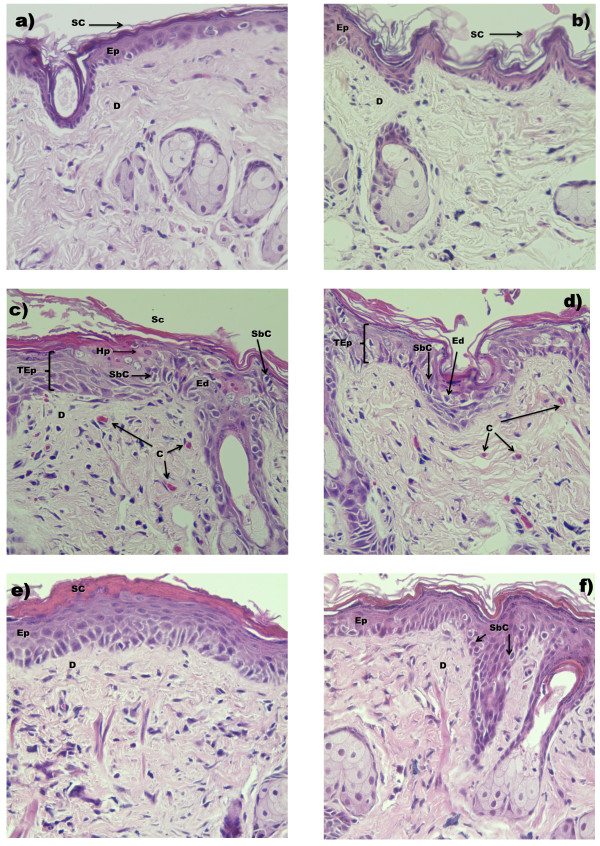
**Histology of skin samples from UVB-irradiated SKH-1 mice treated with BCME.** H&E; 400X magnifications. **a)** U: group untreated; **b)** C-: Negative control (Vehicle 200 μL of ethanol); **c and** **d)** C+UV: Skin without protection (Vehicle and UV) **e)** BCME: *B. cordata* methanolic extract; and **f)** BCME+UV: *B. cordata* methanolic extract and UV. C Vessel congestion; D dermis; ED edema; Ep epidermis; Hp hypertrophy cells; SbC Sunburn cell; SC stratum corneum; TEp Thickening epidermis.

A histological evaluation was performed on the SKH-1 hairless mice exposed to UVB. Figure 4 shows the most representative results of the histological study. The skin of the U, C- and BCME groups showed no histological damage (Figure 4a, b, and e panels). Acute exposure of UVB produced histological changes in the C+UV group: intra-/intercellular edema in the epidermis, number of sunburn cells, thickening of the stratum corneum and epidermis, vessel congestion, and perivascular edema in the dermis (Figure 4c and d). In contrast, skin from BCME+UV mice only showed a slight thickening of the epidermis and did not exhibit the UVB-induced inflammatory changes such as vessel congestion or edema in the epidermis and dermis. However, this group showed fewer numbers of sunburn cells when compared with the C+UV group (Figure 4f). These results correlated with macroscopic observations of erythema, likely because redness is the result of vessel congestion [33]. BCME+UV treated mice showed no edema; thus, it is likely that the effect is due to the presence of linarin in the BCME. One of the main components of BCME is linarin. Martínez-Vázquez, [15] demonstrated the anti-inflammatory properties of this compound. In our study, we also found that the formation of sunburn cells was reduced in the skin of mice treated with BCME+UV, indicating that few keratinocytes of the epidermis were entering the programmed cell death pathway. Previous studies have demonstrated the antioxidant and photoprotective capabilities of verbascoside [20, 31]. The presence of this compound in BCME could explain the reduced presence of sunburned cells on the skin of mice treated with BCME+UV because cells protected from photo-oxidative stress do not initiate an apoptotic pathway. Many agents (such as ultraviolet light filters) affect the transmission of ultraviolet light to human skin. In addition, agents such as antioxidants can modulate the effects of ultraviolet light on the skin. Most of the naturally occurring chemopreventive phenolics exert multifaceted actions, and any clinical applications using these substances should be based on the precise understanding of the physiologically relevant mechanisms of action [2].

### *In vivo*BCME penetration study

To determine the penetration of the active components of BCME into epidermis, we proceeded to perform an experiment known as tape stripping. At equal that experimental protocol; each mouse was treated topically with 2 mg/cm^2^ of the BCME for 15 minutes. Active components of BCME penetrate at 0.82±0.2% (33.4±9.0 μg/cm^2^ in weight) (Table 1). According to this result, the penetration of BCME into the skin could explain the absence of edema and the reduction of sunburn cells. It would think that the amount of active compounds of BCME that penetrates into the skin is low; however the number of molecules able to penetrate is significant according to the Avogadro’s number. Thitilertdecha et al. [34], determined that verbascoside has a low octanol-water partition coefficient (P -0.03), and performed *in vitro* permeation experiments using pigskin. Their results indicated that verbascoside penetrates into the skin (0.270 nmol/cm^2^). Our results suggest SKH-1 mice skins are more permeable to this kind of compounds than pigskin.

### Genotoxicity assay of BCME

In this study, the *Vicia faba* micronucleus test, which detects chromosomal breakage and aneuploidy, was employed to evaluate the genotoxic potential of BCME. These bioassays are sensitive, and it is relatively simple to detect the genotoxic effects of several compounds with them. BCME did not significantly affect the cellular division of the roots and had no clastogenic effects on the cells (data not shown). Thus, BCME extract cannot be considered a mutagenic agent. Further studies should be carried out to evaluate the safety of the compounds present in BCME in other toxicological models.

## Conclusions

UV exposure causes skin damage, and chronic exposure carries a risk of skin cancer. The increase in UVB radiation that reaches the earth’s surface enhances the deleterious effects of this radiation on human health. Therefore, new strategies are needed to combat UV skin damage. The development of promising photoprotective agents requires continuous research throughout the development process. In addition, this study is the first report describing the topical administration of BCME, and it was found to protect against UVB-induced skin damage in SKH-1 mice. These results demonstrate that BCME exhibits absorbance in the UVB spectrum, contains significant scavenging ability for the hydroxyl radical and is not a genotoxic agent in the micronucleus test. The present findings demonstrate that BCME is endowed with good *in vivo* skin photoprotective properties and that this is likely due to the polyphenol content of BCME (verbascoside and linarin). Therefore, these results suggest that BCME may exhibit some potential to prevent photodamage. According to our results, BCME possess sunscreen properties and also it is possible that the BCME compounds inside the skin of mice carry out photochemoprotective activities. Additional studies to better understand the mechanism of action of BCME responsible for its photoprotective effects are in progress.
